# Exploration of the Mechanism of Erchen Decoction in Ameliorating Obesity by Regulating Lipid Deposition *via* the AMPK/SIRT1/PGC-1α Signaling Pathway

**DOI:** 10.2174/0118715303373517250919055421

**Published:** 2025-10-15

**Authors:** Yue Luo, Hao Gao, Zhuo Zhao, Tianshu Gao, Ziyu Liu, Xiao Yang

**Affiliations:** 1 School of Graduate Studies Liaoning University of Traditional Chinese Medicine Shenyang 110847, China;; 2 Department of Endocrinology, Affiliated Hospital of Liaoning University of Traditional Chinese Medicine, Shenyang 110032, China;; 3 Department of Traditional Chinese Medicine Pharmacy, Liaoning Academy of Traditional Chinese Medicine, Second Affiliated Hospital, Liaoning University of Traditional Chinese Medicine, Shenyang 110034, China

**Keywords:** Obesity, erchen decoction, AML-12 cells, lipid deposition, AMPK/SIRT1/PGC-1α signaling pathway, lipid metabolism

## Abstract

**Introduction:**

Obesity, a global health crisis, necessitates innovative therapeutic strategies. Traditional Chinese Medicine (TCM), including Erchen Decoction (ECD), offers potential benefits; however, the molecular mechanisms underlying ECD's anti-obesity effects, particularly on lipid metabolism, remain unclear. This study investigates the mechanism by which ECD improves lipid metabolism through the AMPK/SIRT1/PGC-1α signaling pathway.

**Methods:**

AML-12 hepatocytes were cultured and divided into Control, Model (palmitic acid-induced lipid deposition), and treatment groups (ECD serum: 2%, 4%, 8%; orlistat: 10 µM). Cell viability, lipid accumulation, and triglyceride (TG) content were measured. Key lipid metabolism genes (p-AMPK/AMPK, SIRT1, PPARα, PGC-1α, Nrf2, TFAM) were evaluated using RT-qPCR, Western Blot, and immunofluorescence. Network pharmacology analysis identified ECD's targets and pathways.

**Results:**

The Model group showed increased lipid droplets and TG content, with reduced AMPK, SIRT1, PPARα, PGC-1α, Nrf2, and TFAM expression compared to Control. ECD treatment significantly decreased lipid droplets and TG content (at all doses), with the 4% dose being the most effective. ECD up-regulated all tested genes, confirmed by immunofluorescence (p-AMPK, SIRT1, PGC-1α). Network pharmacology highlighted the overlap between ECD and obesity pathways, with a particular emphasis on lipid metabolism.

**Discussion:**

ECD improves lipid metabolism in AML-12 hepatocytes by activating the AMPK/SIRT1/PGC-1α pathway, consistent with prior research. The upregulation of Nrf2 and TFAM indicates enhanced mitochondrial function and resistance to oxidative stress. Limitations include the use of *in vitro* models; future studies should focus on clinical translation and dosage optimization.

**Conclusion:**

ECD regulates lipid metabolism *via* the AMPK/SIRT1/PGC-1α pathway, providing a scientific basis for its clinical use in obesity-related diseases. This study highlights ECD as a promising therapeutic strategy for improving lipid metabolism and reducing obesity-associated risks.

## 1 INTRODUCTION

Obesity is typically characterized by the excessive accumulation of adipose tissue, which disrupts physiological homeostasis [1]. A BMI threshold of 30 kg/m^2^ or higher serves as the clinical benchmark for diagnosing this condition. Emerging evidence underscores its pathogenic role in elevating morbidities and mortality across diverse populations [2]. The multifactorial etiology of obesity involves complex interactions between dietary behaviors, genetic susceptibilities, and psychosocial stressors in disease pathogenesis [3]. Among these factors, a high-fat diet (HFD) emerges as a primary driver in obesity pathogenesis [4]. During 1993-2015, mainland China experienced a threefold surge in adult overweight prevalence from 4.2% to 15.7% [5]. Projective models further highlight global urgency, as De *et al.* forecast an 84.1% increase in male obesity cases and a 56.0% rise in female cases by 2030 in Belgium [6]. The rising obesity crisis has become a pressing public health concern, requiring innovative, evidence-based intervention strategies.

Adipose tissue undergoes excessive accumulation in obese individuals [7]. This accumulation promotes lipolysis, a process that facilitates the influx of free fatty acids (FFAs) into the liver [8]. When hepatic FFA influx exceeds β-oxidation capacity, lipids ectopically deposit within hepatocytes, triggering hepatic steatosis [9]. This pathological process is linked to impaired β-oxidation, reduced VLDL secretion, and dysregulated DNL, leading to dyslipidemia: elevated TC, TG, and LDL-C, with reduced HDL-C [10]. The liver serves as a central hub for lipid metabolism, maintaining metabolic homeostasis through the tightly coordinated synthesis and catabolism of fatty acids [11]. SREBP-1c acts as a master regulator, activating FAS and ACC genes to promote lipogenesis [12]. FAS, the rate-limiting enzyme in DNL, drives excessive endogenous fatty acid production and lipid deposition [13]. ACC catalyzes the production of malonyl-CoA for FA synthesis, while SCD1 converts saturated to unsaturated FAs for TG, CE, and PL biosynthesis [14]. Notably, silencing SREBP-1c expression attenuates hepatic steatosis and suppresses FAS/ACC pathway activity [15]. Similarly, inhibiting SCD1 activity reduces hepatocyte lipid accumulation [16]. These findings highlight the potential of targeting the SREBP-1c/FAS/SCD1 axis to ameliorate hepatic lipid dysregulation in obesity.

AMPK, a conserved serine/threonine kinase, enhances NAD^+^/NADH ratios to activate SIRT1 [17]. In HFD-induced NAFLD models, dual SIRT1/PPAR-α activation accelerates fatty acid oxidation, alleviating hepatic lipid accumulation [18]. Resveratrol triggers autophagy *via* AMPK-SIRT1, elevating LC3-II to mitigate lipid dysregulation and inflammation [19, 20]. PGC-1α uses a dual strategy: it upregulates NRF2 for fatty acid oxidation and increases mitochondrial number/function *via* TFAM [21, 22]. Notably, PPAR-γ interacts with PPAR response elements (PPREs) in the Nrf2 promoter to activate antioxidant gene transcription [23]. Hepatic SIRT1 deficiency disrupts PPAR-α signaling and fatty acid β-oxidation [24]. Collectively, these findings illuminate a multi-tiered regulatory framework in which the AMPK-SIRT1 axis interfaces with metabolic transcription factors to sustain energetic equilibrium.

The traditional Chinese herbal formula ECD is derived from the classical medical text Taiping Huimin Heji Jufang [25]. Recent pharmacological investigations demonstrate that ECD exerts multifaceted metabolic benefits in HFD-fed rodents. The therapeutic effects encompass lipid-lowering actions, attenuation of oxidative stress, and suppression of inflammatory responses [26]. Specifically, these benefits include amelioration of lipid transporter dysfunction, improved glucose tolerance, stimulated insulin secretion, and reduced serum lipid levels [27]. Notably, activation of AMPK by ECD has been implicated in correcting lipid metabolic dysregulation in obese rats [28]. Despite the documented anti-obesity effects of ECD, the mechanistic role of AMPK/SIRT1/PGC-1α signaling axis regulation remains unclear. This study explores the expression dynamics of pathway components to clarify ECD-mediated metabolic improvements in obesity.

## 2 MATERIALS AND METHODS

### 2.1 Reagents and Materials

Cell culture plates and 96-well cell culture plates were purchased from Nest (704002, 701002); Penicillin/streptomycin solution, 0.25% Trypsin-EDTA, sheep anti-rabbit-HRP, sheep anti-mouse HRP, and ECL substrate solution were purchased from Biyuntian (C0222, C0201, A0208, A0216, P0018S). Fetal calf serum was purchased from Gibco (10270-106); DMEM/F12 medium was purchased from Shanghai Yuanpei (L310KJ). The TG detection kit was purchased from Biosharp (BL1200A). The CCK-8 assay kit, PBS, TRIZOL, and PMSF were purchased from Solebo (CA1210, P1032, R1100, P0100). ToloScript RT EasyMix for qPCR was purchased from TOLOBIO (22106); chloroform, isopropanol, SDS, bromophenol blue, and methanol were purchased from Sinopdrug Group Chemical Reagent Co., LTD. (10006818, 40049962, 30166428, BO449-5G, 10014118). The SYBR qPCR Master Mix was purchased from TOLOBIO (22204-01); DEPC water, Tris-Base, and Tween-20 were purchased from Biosharp (BL510A, BS083, BS100); TEMED was purchased from Phygene (PH0341); the protein marker was purchased from Servicebio (G2058). A PVDF membrane (0.45 µm) was obtained from Millipore (IPVH00010). Primers were designed and synthesized by Saisofei Biotechnology Co., LTD. The Anti-SIRT1 Rabbit pAb was sourced from Wanleibio (WL00599). The polyclonal antibody for Nrf2 was purchased from Jiangsu Baijia Biotechnology Co., LTD. (IPB0035). The PGC1α monoclonal antibody, INS monoclonal antibody, and INSR polyclonal antibody were purchased from Proteintech Company (66369-1-Ig, 66198-1-Ig, 20433-1-AP). The Acetyl-Lysine Mouse Monoclonal Antibody was obtained from Beyotime Biotechnology (AG0433). The SREBP1 antibody, FAS antibody, SCD antibody, ACC antibody, BNIP3 antibody, Beclin 1 antibody, LC3B antibody, Bax antibody, Bcl-2 antibody, Cleaved-Caspase 3 antibody, NR1H3 antibody, RXR alpha antibody, PPARα antibody, AMPK antibody, p-AMPK antibody, TFAM antibody, and GAPDH were purchased from Affinity Company (AF6283, AF5342, DF13253, AF6421, DF8188, AF5128, AF4650, AF0120, AF6139, AF7022, DF6864, DF8459, AF5301, AF6423, AF3423, AF0531, AF7021).

### 2.2 Network Pharmacology Analysis

To decipher the molecular mechanisms underlying ECD's anti-obesity effects, we employed a multi-step systems biology approach. The active compounds and targets of ECD herbs (Pinellia ternata, Citrus reticulata Blanco, Poria cocos, Glycyrrhiza uralensis, Zingiber officinale, and Prunus mume) were identified using ETCM (http://www.tcmip.cn/ETCM/), HERB (http://herb.ac.cn/), and TCMSP databases (https://tcmsp-e.com/), with targets standardized to human proteins *via* the UniProt database (http://www.uniprot. org/). Obesity-related targets were extracted from the human Mendelian target database (OMIM, https://db.idrblab.org/ttd/) and the genetic diseases database DisGeNet (https://www.disgenet.org), standardized through UniProtKB (human, Swiss-Prot ID, high confidence). Overlapping ECD-obesity targets were determined using R software (version 3.5.3, available at https://www.r-project.org) (Venn diagram) and mapped into the STRING database (https://string-db.org)-derived protein-protein interaction (PPI) networks. Functional enrichment analyses, including KEGG pathway and Gene Ontology (GO) terms, were performed on overlapping targets using the DAVID database (https://david.ncifcrf.gov/) (*p*<0.05), with visualizations generated in R (ggplot2). Finally, multi-level networks integrating PPI and herb-compound-target interactions were constructed in Cytoscape to elucidate the pharmacological actions of ECD in obesity.

### 2.3 Animals and treatment

Thirty-six SPF male Sprague-Dawley rats (6-7 weeks, 180-220 g) were purchased from Liaoning Changsheng Biotechnology Company and housed under controlled conditions (22 ± 2 °C, 40-60% humidity) with ad libitum access to food and water. The animal experiments of this study were approved by the Ethics Committee of the Second Affiliated Hospital of Liaoning University of Traditional Chinese Medicine, and the study protocol was ethically reviewed and approved (Record No.: AEC01002-RS01). The high-fat diet (60% fat, D12492) and standard chow (D12450J) were obtained from commercial suppliers. Animals were stratified by body weight and randomized into a normal group (n = 6, standard diet) and a model group (n = 30, high-fat diet). The model cohort was further divided into 5 subgroups (n = 6 each): untreated controls, ECD low/medium/high-dose (0.5445 g·kg^−1^, 1.089 g·kg^−1^, 2.178 g·kg^−1^), and orlistat-treated groups. All groups except the normal group received a high-fat diet throughout the 8-week study. Post-intervention, rats were fasted for 12 h before terminal blood collection *via* the retro-orbital sinus. Sera and livers were processed for biochemical assays and histological/molecular analyses. All procedures adhered to institutional ethical standards.

### 2.4 Establishment of Lipid Accumulation Model

AML12 cells were revived and cultured in DMEM/F12 medium supplemented with 10% fetal bovine serum and 1% penicillin-streptomycin under standard conditions (37°C, 5% CO_2_). Cells were subcultured at 90% confluence, and passages 3-4 were utilized for experiments. Log-phase cells were seeded at 8 × 10^3^ cells/well in 24-well plates for model establishment. After adherence, 0.32 mmol/L palmitic acid was added for 24h to induce steatosis. The experimental groups included a blank control, model control, 10 µM positive control, and 2%, 4%, and 8% ECD treatment groups.

#### 2.4.1 Cell Viability was Determined by CCK-8 Assay

Cells in the logarithmic growth phase were plated in 96-well plates. Following exposure to varying concentrations of drug-containing serum at 37°C with 5% CO_2_, 10μL of CCK-8 solution was added to each well, and the plates were incubated for 3 hours. The absorbance at 450nm was measured by a microplate detector to calculate the cell viability: Cell viability (%) = (A(drug added)-A(blank)) / (A(0 drug added)-A(blank))×100.

### 2.5 Oil Red O Staining

Liver tissues were harvested and dehydrated in sucrose solution, followed by embedding in optimal cutting temperature (OCT) compound. Cryosections were prepared and subjected to Oil Red O staining to visualize lipid droplets. Sections were differentiated in 75% ethanol, counterstained with hematoxylin, and rinsed under running water to enhance contrast. Slides were mounted with glycerin-gelatin and examined under a light microscope to evaluate hepatic morphology, including lipid accumulation and cellular architecture.

### 2.6 Serum Lipid Profiles

The serum levels of TC, TG, LDL-C, and HDL-C in rats were measured using an automatic biochemical analyzer following the manufacturer's instructions provided in the assay kit.

### 2.7 Quantitative Real-time PCR

Total RNA was extracted from 20 mg of murine liver using a commercial spin column kit following the manufacturer’s protocol. RNA concentration was determined, adjusted to 200 ng/μL, and reverse-transcribed into cDNA. Quantitative PCR (qPCR) was carried out under standard cycling conditions (95°C for 10 min, followed by 40 cycles of 95°C for 15 s and 60°C for 1 min). Relative mRNA expression was calculated using the 2^-ΔΔCt method. Gene-specific primers (Table **1**) were synthesized by Sangon Biotech (Shanghai, China).

### 2.8 Western Blot

Total protein was isolated from 100 mg of rat liver tissue and quantified *via* the BCA assay. Equal protein loads were resolved by SDS-PAGE (200 V) and transferred to PVDF membranes (110 V, 70 min). Membranes were blocked with 5% non-fat milk, probed with primary antibodies overnight at 4°C, and incubated with secondary antibodies at room temperature. Immunoreactive bands were detected using enhanced chemiluminescence (ECL) and quantified by densitometry using ImageJ software.

### 2.9 Immunofluorescent Staining

AML-12 cells under optimal growth were plated on 96-well glass slides, assigned to experimental groups, and incubated for 24 h at 37°C. Samples were fixed in 4% paraformaldehyde, blocked with 3% BSA, and incubated overnight at 4°C with primary antibodies (1:500/1:4000 dilution). A secondary antibody (goat anti-rabbit, 40-50 µL) was applied for 1 hour at 37°C, followed by PBST washes. Nuclei were counterstained with DAPI, and fluorescence intensity was quantified using ImageJ after imaging on a confocal microscope.

### 2.10 Co-Immunoprecipitation

Lysate (1 mL) was incubated with isotype control antibody (5 μL) on ice for 1 h, followed by Protein A/G PLUS-Agarose (100 μL) addition and rotation at 4°C for 20 min. Post-centrifugation, the supernatant was collected. Total protein (500 μg) in RIPA buffer (450 μL) was combined with excess primary antibody and inhibitor-containing RIPA to 0.5 mL, incubated overnight at 4°C. Protein A/G PLUS-Agarose (70 μL) was added, rotated for 4 h, washed, centrifuged, and pellet resuspended in loading buffer (20 μL). The sample was boiled for 3 min and utilized for WB.

### 2.11 Statistical Analysis

Data were analyzed using SPSS 23.0, with results presented as mean ± standard deviation (x ± s). Prior to data analysis, tests for normality and homogeneity of variance were conducted. A t-test was employed to assess differences between two groups, while a one-way ANOVA was utilized for comparisons among multiple groups. Statistical significance was set at *P* < 0.05.

## 3 RESULTS

### 3.1 Network Analysis and Pathway Enrichment

The network analysis revealed distinct clusters of herbs, diseases, and biological pathways (Fig. **1A**). Notably, the Venn diagram (Fig. **1B**) showed an overlap of 402 nodes between herbs and diseases, indicating shared biological targets. (Fig. **1C**) showed the PPI Network of Cross-Linked Targets. Pathway enrichment analysis (Fig. **1D**) highlighted significant responses, including “response to lipopoly saccharide” and “lipid and atherosclerosis,” suggesting a crucial role for lipid metabolism in the therapeutic effects of the herbs. Additionally, hierarchical clustering (Fig. **1E**) identified key ontologies, such as “AGE-RAGE signaling pathway in diabetic complications,” further emphasizing the multifaceted biological impacts of the studied herbs.

### 3.2 Histopathological and Biochemical Analysis

The hepatic sections stained with H&E showed varying degrees of tissue morphology across different groups (Fig. **2A**). A quantitative assessment of serum biochemical parameters revealed significant differences in TG, TC, HDL-C, LDL-C, AST, and ALT levels among the groups (Fig. **2B**). Notably, the HFD group exhibited higher lipid profiles and liver enzyme levels compared to the control, indicating potential liver dysfunction.

### 3.3 Effects of Erchen Decoction-Containing Serum on AML-12 Cells

ECD-containing serum reduced hepatic lipid accumulation in AML-12 cells after 24 h of treatment. The CCK-8 assay (Fig. **3A**) confirmed no significant cytotoxicity at concentrations of 2%, 4%, and 8%. Oil Red O staining (Fig. **3B**) demonstrated a marked reduction in lipid droplet accumulation in ECD-treated groups compared with the model group. Consistently, intracellular TG levels (Fig. **3C**) were significantly decreased, with the greatest reduction observed in the orlistat group.

### 3.4 Insulin Resistance-Related and Apoptosis-Related Gene Expression in AML-12 Hepatocytes Following ECD Treatments

Western blot analysis revealed significant changes in the expression of insulin resistance-related proteins (Fig. **4A**) and apoptosis-related proteins (Supplementary Fig. **1A**) in AML-12 hepatocytes following ECD treatments. For insulin resistance-related markers, INS, INSR, NR1H3, and RXRα protein levels were modulated by ECD treatments, with notable differences compared with the control and model groups (Figs. **4B**-**E**). For apoptosis-related markers, Bax, Bcl-2, and cleaved caspase-3 protein levels were significantly altered, indicating the impact of ECD treatments on apoptotic pathways (Supplementary Figs. **1B**-**D**).

### 3.5 Insulin Resistance-Related and Apoptosis-Related Gene Expression in AML-12 Hepatocytes Following EC Treatments

Western blot analysis demonstrated significant changes in the expression of lipid metabolism-related proteins (Fig. **5A**) and autophagy-related proteins (Supplementary Fig. **2A**) in AML-12 hepatocytes following ECD treatments. For lipid metabolism, SREBP-1c, ACC, FAS, and SCD1 protein levels were notably altered, with significant differences compared with the control and model groups (Figs. **5B** - **E**). For autophagy-related markers, BNIP3, Beclin-1, and LC3-I/II protein levels were significantly modulated, indicating the impact of ECD treatments on autophagic pathways (Supplementary Figs. **2B** and **D**).

### 3.6 ECD-Containing Serum Can Improve Lipid Deposition in AML-12 Hepatocytes Through AMPK/SIRT1/PGC-1α Pathway

In AML-12 hepatocytes, the model group exhibited a significant reduction (*p* < 0.01) in the mRNA and protein expression of AMPK, SIRT1, PPARα, PGC-1α, Nrf2, and TFAM compared with the control group (Figs. **6A** and **B**). The mRNA expression levels of AMPK, SIRT1, PPARα, PGC-1α, Nrf2, and TFAM were significantly elevated in the low-, medium-, and high-dose ECD groups, as well as in the western medicine group, compared with the model group.

### 3.7 ECD Containing Serum can Improve Lipid Deposition in AML-12 Hepatocytes by Enhancing the Fluorescence Intensity of AMPK, SIRT1, And PGC-1α Pathways

Immunofluorescence analysis revealed a significant reduction in p-AMPK, SIRT1, and PGC-1α expression in the model group compared with the control group (Figs. **7A** and **B**). This reduction was reversed in the low-, medium-, and high-dose ECD groups, as well as in the western medicine group.

### 3.8 SIRT1-Mediated Deacetylation of PGC-1α Following EC Treatments

Western blot analysis revealed the effects of inhibitor treatments on SIRT1 and the deacetylation of PGC-1α in AML-12 hepatocytes. The representative Western blot analysis (Fig. **8A**) showed changes in protein expression levels of SIRT1, PGC-1α, and acetylated-Lys PGC-1α. Densitometric quantification (Figs. **8B**-**F**) demonstrated significant alterations in these proteins, normalized to GAPDH.

## 4 DISCUSSION

In recent years, with the increasing incidence of diseases associated with abnormal lipid metabolism, the effective regulation of lipid metabolism has become a research focus. Traditional Chinese Medicine (TCM), as the traditional medical practice of China, has demonstrated significant potential in modulating lipid metabolism due to its unique theoretical framework and extensive clinical experience.

The understanding of fat in TCM has a long history. In the Lun on Hygiene Disorders of Ling Shu, it is stated that “people have fat, ointment, and meat.” When the spleen and stomach are deficient, the diet is improper, or excessive consumption of fatty, rich, and greasy foods occurs, phlegm and dampness are generated internally, which then transform into fat. Therefore, TCM believes that phlegm dampness is the main pathogenesis of abnormal lipid metabolism. ECD is a classic Chinese prescription for treating phlegm. It is derived from the Taiping Huimin Huijianxian Bureau Prescription. In the prescription, Pinellia triticum is used to dispel phlegm, tangerine peel is used to promote qi, and Poria is used to benefit the water. This recipe has the effect of invigorating the spleen and dissipating phlegm, and regulating qi. In recent years, clinical studies have demonstrated that ECD can significantly improve abnormal lipid metabolism in patients with non-alcoholic fatty liver disease, hyperlipidemia, and other metabolic disorders, and reduce blood lipid levels in patients [29]. Additionally, network pharmacology studies have shown that ECD can improve obesity-related diseases by regulating lipid metabolism and reducing adipose tissue inflammation [30]. In this study, compared with the Control group, the lipid droplets and TG content in the Model group increased significantly. Compared with the Model group, the lipid deposition and TG content in each dose of the TCM group and the western medicine group were down-regulated, with the middle dose showing the most significant reduction. These results suggest that ECD can enhance lipid metabolism and reverse the abnormal state of lipid metabolism in hepatocytes.

AMPK, SIRT1, and PGC-1α are key molecules involved in the regulation of energy and lipid metabolism [31]. As a cellular energy sensor, AMPK is activated under energy-deficient conditions to promote ATP production and inhibit ATP-consuming processes, thereby maintaining energy balance [32]. SIRT1 is a deacetylase that regulates diverse biological processes, including metabolism, stress response, and aging, by deacetylating various substrates such as transcription factors and metabolic enzymes [33]. PGC-1α is a central regulator of mitochondrial biogenesis and oxidative metabolism, promoting fatty acid oxidation and mitochondrial function in cooperation with AMPK and SIRT1 [34]. In this study, ECD effectively inhibited palmitic acid (PA)-induced lipid deposition and significantly reduced triglyceride (TG) levels in AML-12 cells. This effect is closely associated with the activation of the AMPK/SIRT1/PGC-1α signaling pathway. Specifically, compared with the model group, orlistat treatment significantly increased the mRNA and protein expression levels of p-AMPK/AMPK, SIRT1, PPARα, PGC-1α, Nrf2, and TFAM. These results suggest that ECD-containing serum may enhance the deacetylase activity of SIRT1 through AMPK activation, ultimately upregulating PGC-1α and its downstream target genes, thereby promoting fatty acid oxidation and reducing lipid deposition.

Recently, multiple studies have confirmed the importance of the AMPK/SIRT1/PGC-1α signaling pathway in regulating lipid metabolism. Relevant studies have shown that activated AMPK can effectively act on downstream target proteins to improve cellular lipid metabolism and hepatocyte function by reducing oxidative stress, enhancing cell autophagy, and modulating the inflammatory response [35, 36]. Activation of the AMPK/SIRT1/PGC-1α pathway can improve glucose and lipid metabolism disorders, thereby reducing associated inflammatory responses [37]. Aerobic exercise-induced activation of this pathway has also been shown to lower obesity-related elevations in blood lipids [38]. In the present study, ECD-containing serum significantly upregulated the expression of SIRT1 and PGC-1α, suggesting that ECD may improve lipid metabolism through a similar mechanism. Price *et al.* further reported that AMPK activation increases fatty acid oxidation and reduces lipid accumulation [39]. Consistently, in the present study, ECD-containing serum significantly increased the p-AMPK/AMPK ratio, suggesting that AMPK activation may be a key mechanism by which ECD inhibits lipid deposition. These findings are consistent with those of Price *et al.* and further  support  the hypothesis that ECD enhances lipid metabolism through the AMPK/SIRT1/PGC-1α signaling pathway. In addition, upregulation of Nrf2 and TFAM was also observed. Nrf2 is a key regulator of antioxidant defense and mitochondrial function [40], whereas TFAM is essential for mitochondrial DNA replication and transcription [41]. The ability of ECD-containing serum to upregulate Nrf2 and TFAM may therefore improve mitochondrial function and strengthen resistance to oxidative stress, further promoting fatty acid oxidation and reducing lipid deposition. This observation is consistent with previous studies on the roles of Nrf2 and TFAM in metabolic regulation, offering a new perspective on how ECD contributes to the improvement of lipid metabolism.

ECD, as a traditional Chinese medicine formula, has a complex composition and contains a variety of active ingredients. Although the specific components of ECD were not isolated and identified in this study, combined with existing studies, it can be speculated that the effect of ECD on improving lipid metabolism may result from multiple components and multiple targets. These components may act synergistically to activate the AMPK/SIRT1/PGC-1α signaling pathway, thereby improving lipid metabolism. In addition, this study also found that a middle dose of ECD containing serum was the most effective in inhibiting lipid deposition and upregulating the expression of related genes. These results suggest that the effect of ECD-containing serum may be dose-dependent, and an appropriate dose may be more effective in exerting its efficacy. Future studies can further investigate the detailed effects of different doses of ECD-containing serum on lipid metabolism to optimize its clinical application.

While this study has elucidated the potential mechanisms by which ECD regulates lipid metabolism, certain limitations remain. First, the study mainly focused on *in vitro* cell experiments and animal models, which restricts its direct applicability and validation in humans. Second, the specific active components of ECD were not fully isolated or identified, making it difficult to determine their precise targets and mechanisms of action. Future studies should emphasize clinical translation, further investigate the active components and underlying mechanisms, and comprehensively evaluate the efficacy of ECD in regulating lipid metabolism.

## CONCLUSION

This study reveals the potential mechanism by which ECD regulates lipid metabolism, indicating its remarkable effect in improving abnormal lipid metabolism. The mechanism behind this may be related to the upregulation of the AMPK/SIRT1/PGC-1α signaling pathway. This study provides a scientific basis for optimizing ECD in clinical applications and outlines the direction for future research.

## STUDY LIMITATIONS

This study reveals partial mechanisms by which ECD ameliorates obesity, yet several limitations remain. The experiments were primarily conducted using AML-12 cell models and high-fat diet-induced animal models; therefore, the direct applicability of these findings to humans requires further validation. Moreover, due to the complex composition of Erchen Decoction, the specific active ingredients were not isolated or identified in this study, making it difficult to pinpoint the exact material basis and target mechanisms responsible for its effects. Future research should prioritize clinical translation, further investigate the active constituents, and validate the efficacy and safety of ECD in human trials.

## Figures and Tables

**Fig. (1):**
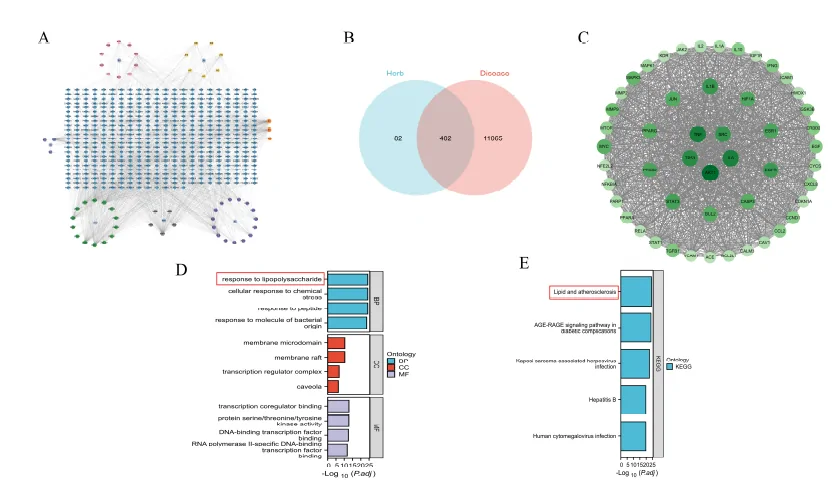
Network pharmacology analysis. (**A**) Compound-target interaction network. (**B**) Intersection of ECD with obesity-related targets. (**C**) PPI network of cross-linked targets. (**D**) Gene ontology (GO) biological process enrichment analysis. (**E**) KEGG Pathway enrichment analysis.

**Fig. (2):**
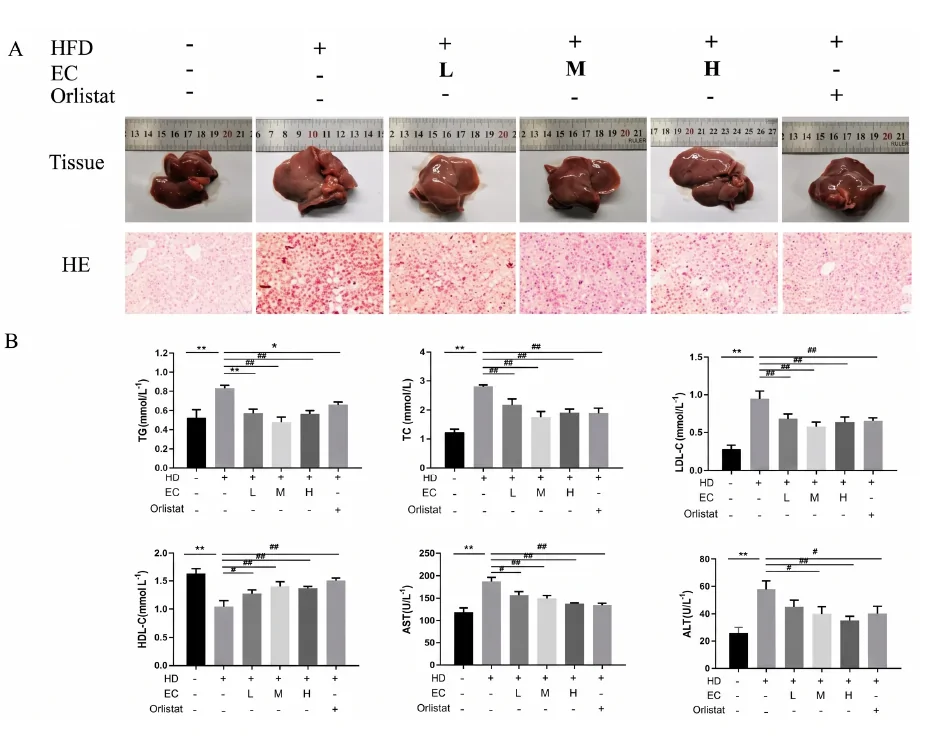
Histopathological analysis of liver tissue and plasma lipid profiles .(**A**) Representative photomicrographs of hepatic sections and H&E staining (**B**) Quantitative assessment of serum biochemical parameters. **P < 0.01 *vs*. control group; ^#^*P* < 0.05 and ^##^*P* < 0.01 *vs* model group.

**Fig. (3):**
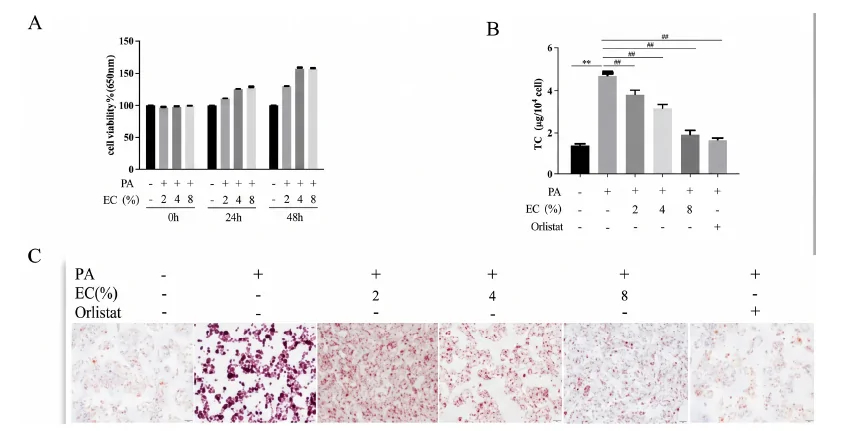
Comprehensive Analysis of cellular responses to metabolic stressors and the intervention effects of ECD. (**A**) Time-dependent cytoprotection by ECD against palmitic acid (PA)-induced toxicity. (**B**) Orlistat-mediated suppression of PA-induced lipid accumulation by ECD. (**C**) Morphological evidence of lipotoxicity mitigation by ECD. **P < 0.01 *vs* control group. ^##^*P* < 0.01 *vs* model group.

**Fig. (4):**
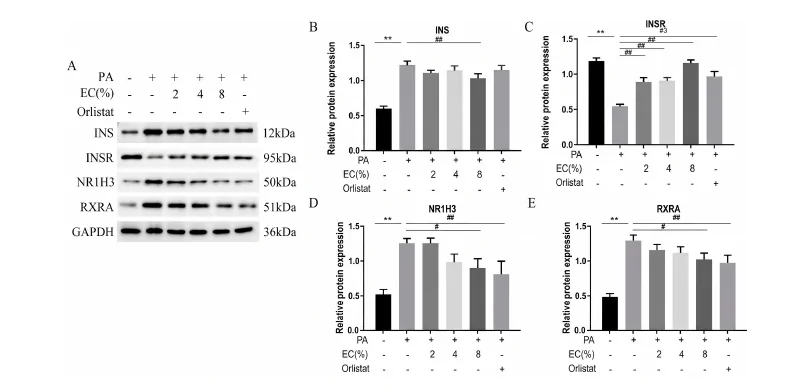
Western blot analysis of insulin resistance-related gene expression in AML-12 hepatocytes following EC treatments. (**A**) Representative western blot analysis showing INS, INSR, NR1H3, RXRA and GAPDH protein levels in AML-12 cells. (**B**) Relative INS protein expression. (**C**) Relative INSR protein expression. (**D**) Relative NR1H3 protein expression. (**E**) Relative RXRA protein expression. **P < 0.01 *vs* control group; ^#^*P*< 0.05 and ^##^*P* < 0.01 *vs* model group.

**Fig. (5):**
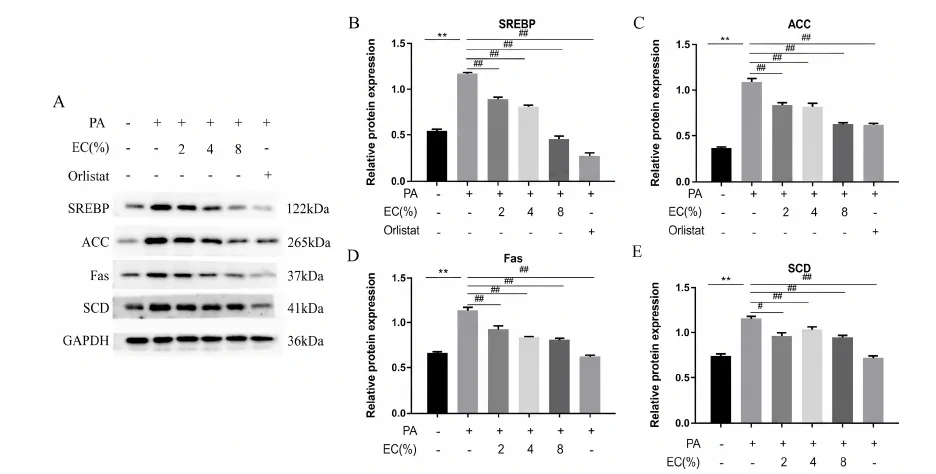
Western blot analysis of lipid metabolism-related gene expression in AML-12 Hepatocytes Following EC Treatments. (**A**) Representative western blot analysis showing SREBP, ACC, Fas, SCD and GAPDH protein levels in AML-12 cells. (**B**) Relative SREBP protein expression. (**C**) Relative ACC protein expression. (**D**) Relative Fas protein expression. (**E**) Relative SCD protein expression. **P < 0.01 *vs* control group; ^#^*P* < 0.05 and ^##^*P* < 0.01 *vs* model group.

**Fig. (6):**
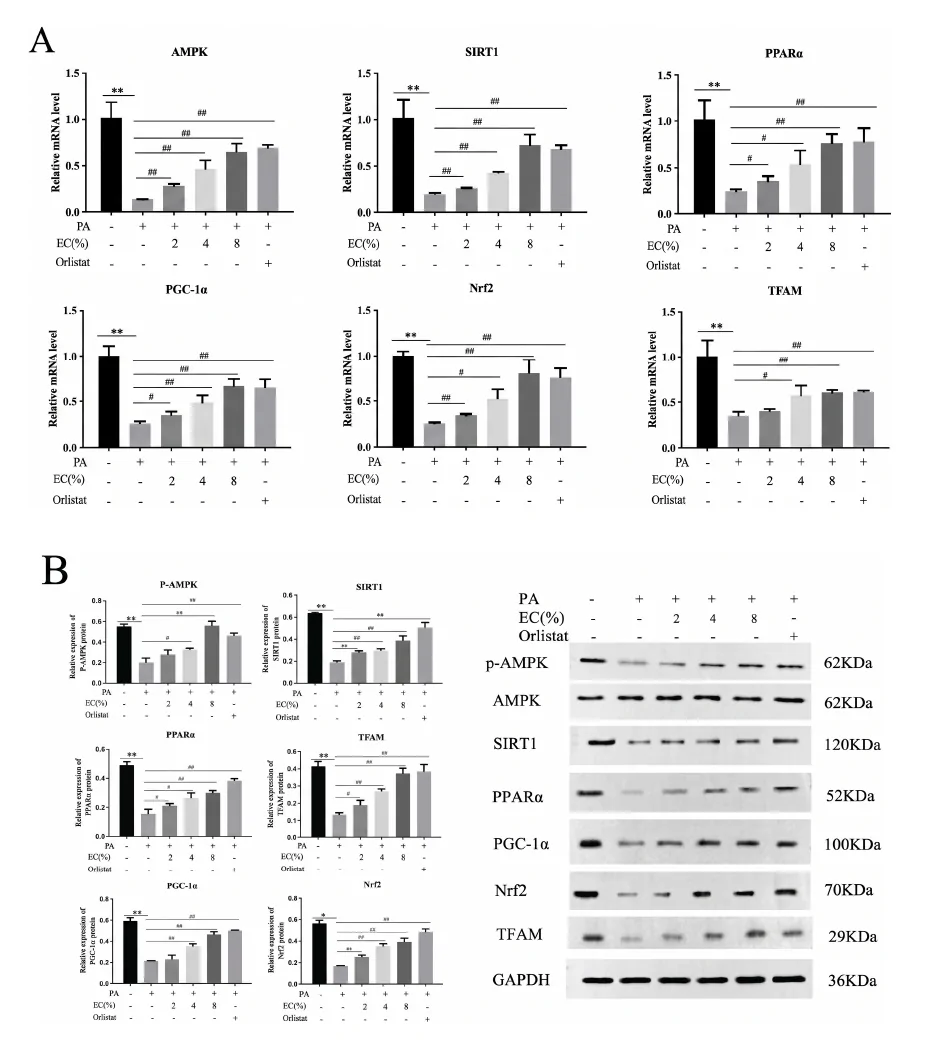
Relative mRNA and protein expression of AMPK, SIRT1, PPARα, PGC-1α, Nrf2, and TFAM in AML-12 hepatocytes. (**A**) Representative RT-qPCR analysis showing AMPK, SIRT1, PPARα, PGC-1α, Nrf2, and TFAM mRNA levels in AML-12 cells. (**B**) Representative western blot analysis showing AMPK, p-AMPK, SIRT1, PPARα, PGC-1α, Nrf2, TFAM and GAPDH protein levels in AML-12 cells. **P < 0.01 *vs* control group; ^#^*P* < 0.05 and ^##^*P* < 0.01 *vs* model group.

**Fig. (7):**
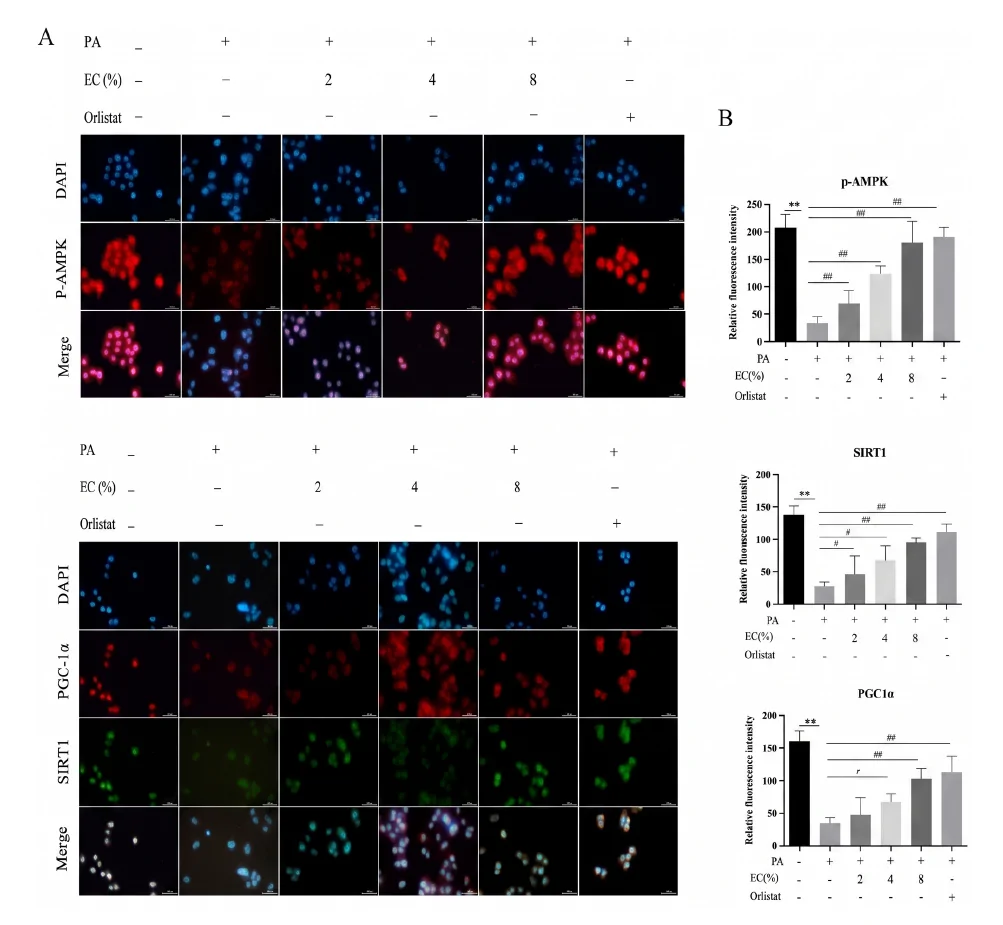
Immunofluorescence analysis of p-AMPK, SIRT1, and PGC-1α expression in AML-12 hepatocytes. (**A**) Representative immunofluorescence staining of each group (×400; scale bar = 100 μm). (**B**) Quantitative fluorescence intensity analysis. **P < 0.01 *vs* control group; ^#^*P* < 0.05 and ^##^*P* < 0.01 *vs* model group.

**Fig. (8):**
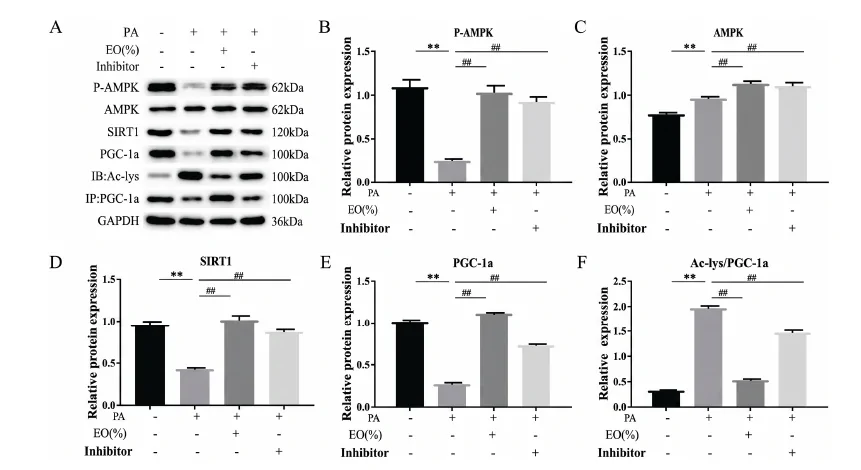
Western blot analysis of the effect of SIRT1 on PGC-1α deacetylation in AML-12 hepatocytes following inhibitor treatment. (**A**) Representative western blot analysis. (**B**) Relative p-AMPK protein expression. (**C**) Relative AMPK protein expression. (**D**) Relative SIRT1 protein expression. (**E**) Relative PGC-1α protein expression. (**F**) Relative acetylated PGC-1α (Ac-lys/PGC-1α) protein expression.Panels (**B-F**) present densitometric quantification of western blot signals normalized to GAPDH.

**Table 1:** Demographic and clinical characteristics of patients with subclinical hypothyroidism.

Primer Name	-	Primer Sequence (5’-3’)
PPARα	F	ACTGCCGTTTTCACAAGTGC
	R	CAGAACGGCTTCCTCAGGTT
AMPK	F	ACCTGAGAACGTCCTGCTTG
	R	TGTTGTACAGGCAGCTGAGG
SIRT1	F	TGAAGCTGTCCGGATTCAGG
	R	CGGTGCTGACTCCTCACATT
PGC-1α	F	CTTTCCCTGGAGGCTCTTGG
	R	TCCCACCCAGATTCAATGGC
NRF2	F	TTGCCCTAGCCTTTTCTCCG
	R	TGGACTCTGAAGGCATCGAG
TFAM	F	CACCACACCACTGAGCAGAT
	R	TATCGCTGTAGTGCCTGCTG
GAPDH	F	GAAGGTCGGTGTGAACGGAT
	R	AGTGATGGCATGGACTGTGG

## Data Availability

The data and supportive information are available within the article.

## LIST OF ABBREVIATIONS

TCM: Traditional Chinese Medicine
ECD: Erchen Decoction
G: Triglyceride
FAs: Free Fatty Acids
AMPK: Adenosine Monophosphate-Activated Protein Kinase
SIRT1: Sirtuin 1
PPARα: Peroxisome Proliferator-Activated Receptor Alpha
PGC-1α: Peroxisome Proliferator-Activated Receptor Gamma Coactivator 1-Alpha
ATP: Adenosine Triphosphate
Nrf2: Nuclear Factor Erythroid 2–Related Factor 2
TFAM: Mitochondrial Transcription Factor A
